# Hospital Staff Appointments

**Published:** 1914-02-07

**Authors:** 


### The Hospital

The Workers' Newspaper of Administrative Medicine and Institutional Life, Administration, National Insurance and Health.

No. 1441, Vol. LV. SATURDAY, FEBRUARY 7, 1914. PRICE ONE PENNY

### HOSPITAL STAFF APPOINTMENTS.

A letter which appeared in our correspondence columns last week (p. 488) raises some very interesting points connected with the tenure of appointments to the visiting staffs of London voluntary hospitals. As our correspondent quite truly pointed out, the ambition of every would-be consultant is to gain such an appointment at a general hospital; or rather, as he might preferably have put it, at a teaching hospital. Now the reason of this ambition is that such posts lead most naturally to that worldly success in private practice to which most of the cleverest young men in the medical profession ultimately look forward. Be it noted in passing that these appointments by no means inevitably mean success in private consulting practice, and that marked success can be and frequently is attained by those who do not hold and never have held them. Be it noted also that some of those who hold these appointments are not ambitious of material success, preferring the pursuit of science to the favours of mammon.

These vacancies at teaching hospitals occur at somewhat long and frequently capricious intervals, and are keenly contested. It follows that there are always a good many able young doctors waiting to compete for one, and anxious to improve their chance by obtaining experience and knowledge in the particular kind of work they intend to take up. Such young physicians, surgeons, gynæcologists, and specialists of many kinds frequently seek, and obtain, election to the staff of special hospitals (or smaller general hospitals) during their period of waiting. If they are ultimately elected to the staff at a teaching hospital, they sometimes resign the post or posts which they may be holding at smaller hospitals; but more often they retain them, and serve two or three, or even occasionally four, institutions. Objections have been raised at times to this system, on the ground that a man can give proper attention to his work at only one hospital at a time; and it has even been argued that pluralism of this sort should be discouraged or forbidden. But until lately no definite policy has been followed, either by institutional managers or by the consultants themselves as a whole; and pluralism has been the order of the day.

Now, however, according to our correspondent, some of the general hospitals are adopting the line of requiring those who obtain visiting appointments to resign all other such posts. The consequences of such a requirement are likely to be momentous. One result, as he points out, is likely to be retaliation by the special hospitals, which naturally resent being made use of as stepping-stones to advancement by the junior members of their staffs. Now the only effective form of retaliation open to them, unfortunately, involves the selection of the comparatively less fit applicants for staff posts; for it is stated that some of the election committees of the lesser charities reject such candidates as appear most likely to succeed at their own mother hospitals. This merely amounts to cutting off the nose to spite the face, and is not a policy which can be recommended. For it means, undoubtedly, that those hospitals which adopt it will in the end find themselves with a second-rate staff, though certainly they will have less frequent changes.

What, then, are the smaller general hospitals and the special hospitals to do in the matter? The problem may become serious and pressing, though we do not believe that it is so at present. For one thing, they can welcome men from the provinces, from Scotland, Wales, and Ireland: as indeed they have always been willing to do, for the fine impartiality of the special hospitals of London has long been recognised, and the cream of extra-metropolitan medical talent has a persistent habit of gravitating to London. For another thing, they can devote much greater care and thought to the selection of their staff than many of them at present do. We could quote special hospitals in London where the staffs are first-class all through, and others where they are quite the reverse. These variations are not fortuitous; they are the result of carefulness and casualness respectively over the filling of staff vacancies. Yet again, they can do much to maintain a high standard by the adoption of the five years' rule, or some similar arrangement whereby all staff appointments are subject to review at regularly recurring intervals of time. To eliminate the weaker vessels by such a method of revision requires courage, honesty, forethought, and a single eye to the interests of the institution; but it is a most valuable reform which those hospitals where it has been adopted would be exceedingly loath to abandon.

There remains the standpoint of the large hospitals which are said to be making the stipulation whose effects have been discussed. Whether they are entirely wise in making a hard-and-fast rule of this sort we question very seriously. It is certainly true that the big general hospitals require nowadays so much of their visiting staffs' energies and time that the consultants may reasonably be expected to put other institutions second; but that is far from the same as insisting on absolute withdrawal from all other institutional work in all circumstances. Admittedly, pluralism has often been overdone; yet the great general hospitals must remember that a policy of selfishness on their part will militate against the interests of the smaller hospitals, the special hospitals, and their patients. Nor do we believe that in the long run it will benefit the large hospitals themselves, which have much to gain by the continued efficiency of the staffing of the smaller and the special institutions.

